# Adjunctive Nebulized Antibiotics: What Is Their Place in ICU Infections?

**DOI:** 10.3389/fmed.2019.00099

**Published:** 2019-05-08

**Authors:** Michael S. Niederman

**Affiliations:** Weill Cornell Medical Center and Weill Cornell Medical College, New York, NY, United States

**Keywords:** nosocomial pneumonia, ventilator-associated pneumonia, aerosol therapy, antibiotics, multi-drug resistant pathogens, clinical trial design, antimicrobial stewardship

## Abstract

Inhaled antibiotics have been used as adjunctive therapy for patients with pneumonia, primarily caused by multidrug resistant (MDR) pathogens. Most studies have been in ventilated patients, although non-ventilated patients have also been included (but not discussed in this review), and most patients have had nosocomial pneumonia. Aerosolized antibiotics are generally added to systemic therapy, and have shown efficacy, primarily as salvage therapy for failing patients and as adjunctive therapy after an MDR gram-negative has been identified. An advantage to aerosolized antibiotics is that they can achieve high intra-pulmonary concentrations that are potentially effective, even for highly resistant pathogens, and because they are generally not well-absorbed systemically, it is possible to avoid some of the toxicities of systemic therapy. When using inhaled antibiotics, it is essential to choose the appropriate agent and the optimal delivery method. Animal and human studies have shown that aerosolized antibiotics reach higher concentrations in the lung than systemic antibiotics, but that areas of dense pneumonia may not receive as much antibiotic as less affected areas of lung. Optimal delivery in ventilated patients depends on device selection, generally with a preference for vibrating mesh nebulizers and with careful attention to where the device is placed in the ventilator circuit and how the delivery is coordinated with the ventilator cycle. Although some studies have shown a benefit for clinical cure, adjunctive therapy has not led to reduced mortality. In some studies, adjunctive aerosol therapy has reduced the duration of systemic antibiotic therapy, thus serving to promote antimicrobial stewardship. Two recent multicenter, randomized, double-blinded, placebo-controlled trials of adjunctive nebulized antibiotics for VAP patients with suspected MDR gram-negative pneumonia were negative for their primary endpoints. This may have been related to trial design and execution and the lessons learned from these studies need to be incorporated in any future trials. Currently, routine use of adjunctive aerosolized therapy cannot be supported by available data, and this therapy is only recommended to assist in the eradication of highly resistant pathogens and to be used as salvage therapy for patients failing systemic therapy.

Inhaled antibiotics have been studied as adjunctive therapy for patients with ventilator associated pneumonia (VAP), hospital-acquired pneumonia (HAP), and severe pneumonia in ventilated ICU patients, particularly when caused by multi-drug resistant (MDR) gram-negative bacteria. The appeal of this type of treatment is the direct delivery of antibiotics to the lower respiratory tract, achieving higher levels of most antibiotics than can be achieved by systemic administration. Although there has been great interest in aerosolized antibiotics for nearly 50 years, there has been a recent resurgence of interest as the result of better small particle delivery systems and also the need to provide effective therapy for MDR pathogens in VAP patients, that are often difficult to eradicate with systemic therapy (1). Conceptually, inhaled antibiotics can be applied when traditional therapy is failing, or they can be used as routine adjunctive therapy at the time of initiating systemic therapy. Recently, two large multi-center randomized trials have tried the latter approach for patients at risk of MDR gram-negative pathogen pneumonia, with disappointing results (2, 3). This review explores the background of aerosolized antibiotics for ICU patients, the methods of delivery, and recent data with inhaled aminoglycosides and other agents.

## Why Inhaled Antibiotics in the ICU?

Inhaled antibiotics have been used in the past to treat bacterial pneumonia, bronchitis, bronchiectasis, viral pneumonia, *Pneumocystis* pneumonia, and tuberculosis. This discussion focuses on severe pneumonia in the ICU, primarily in patients with either VAP or severe CAP needing mechanical ventilation. Although aerosol therapy can be used in non-ventilated ICU patients, the focus of this review is on ventilated patients, since there are not good studies in non-ventilated patients, with documentation of lung deposition. When used in this patient population, the assumption is that the drug is delivered uniformly to both lungs, and effectively to proximal airways and distal aleveoli, and that the antibiotic can penetrate into pneumonic areas of the lung. In animal models, not all of these goals have been achieved (4).

When selecting an antibiotic for aerosol delivery, several desirable features should be considered: the agent should be soluble in solution and able to be delivered as an aerosol; it should kill bacteria in an concentration-dependent fashion; it should remain active in the lung and not be degraded by the delivery process; it can penetrate into sputum and lung secretions; it has minimal systemic absorption, thus minimizing some of the toxicity that can occur with systemic administration; it is not injurious to the airway surface; and ideally it is an antibiotic that does not ordinarily reach high levels in the lung with systemic administration (5). Other desirable features include: an agent that is preservative free, pH adjusted (between 4 and 8), tonicity and osmolality adjusted and with a particle size of 3–5 microns (6). As discussed below, there are ways to optimize delivery with modern devices, but patient factors can also influence delivery and these include: degree of baseline underlying airway and parenchymal lung disease; type of pneumonia (consolidated vs. bronchopneumonia); tidal volume used and mode of mechanical ventilation and ventilator settings (7, 8).

Early studies used aerosolized antibiotics in ICU patients as salvage therapy for patients failing systemic therapy. Michalopoulos et al. reported in 2005 the use of aerosolized colistin with systemic therapy in 8 patients with either *Acinetobacter* spp or *P. aeruginosa* infection, and were able to achieve clinical cure and bacteriologic eradication in most (9). In 2008, Palmer et al. studied aerosolized gentamicin, vancomycin, or both (depending on sputum Gram stain) in patients with ventilator-associated tracheobronchitis (VAT) and reported that after therapy, fewer patients had signs of nosocomial pneumonia, and that more patients were weaned than in the placebo group (10). In 2010, Kofteridis et al. reported data about adjunctive inhaled colistin in 43 patients with VAP due to MDR gram-negatives, and observed a trend to more clinical cure with no difference in eradication, mortality or renal dysfunction, compared to matched controls (11). In 2013, Tumbarello et al. studied 104 patients with VAP caused by colistin-only sensitive gram-negative pathogens, and found that adjunctive inhaled colistin led to higher clinical cure and fewer days of ventilation after pneumonia, than a matched cohort who did not get aerosol therapy (12). While all these reports led to encouragement about using aerosolized therapy, there were other negative studies, and the inconsistency of the data was likely related to a lack of standardization for aerosol delivery methods, or for attention to how delivery was coordinated with the ventilator (13–16).

Animal studies have demonstrated that aerosol therapy can deliver antibiotics to the lung in higher concentrations than when systemic antibiotics are administered (4). Goldstein et al. studied piglets with *E. coli* bronchopneumonia, and showed that aerosolized amikacin delivered by ultrasonic nebulizer into the inspiratory limb of the ventilator circuit, led to 38% of the nebulized dose being retained, and that lung tissue concentrations were 3–30 times higher than when systemic administration was used. Areas of lung with the most severe pneumonia, had lower concentrations after aerosol delivery than less severely affected lung, but still tissue levels were higher than with systemic administration, even in the most severely affected areas. In a study from 2012, Lu et al. demonstrated that patients successfully treated for gram–negative VAP with aerosolized colistin had an increase in thoracic gas volume after therapy, suggesting that the aerosol did reach the distal areas of infection (7).

Based on all of these studies, it is clear that aerosolized therapy can have benefits in the ICU, but that therapy must be given by effective delivery methods. Sole-Llonart et al. recently published a meta-analysis that emphasized these points (17). They evaluated 11 studies, of which 6 were randomized, and 5 were of small size. They found that aerosolized therapy generally involved colistin or aminoglycosides, and led to higher resolution rates for patients with resistant pathogens, but not in those with sensitive pathogens. In addition, aerosol therapy may have prevented the emergence of resistance in patients with VAT and in those with VAP due to susceptible pathogens. However, they also saw an increased risk of respiratory complications in those with baseline severe hypoxemia, with aerosol therapy worsening hypoxemia. As pointed out in the review, there was no consistency regarding which delivery device was used, with some studies not even mentioning mode of delivery, and no consistency to the particle size delivered.

An ICU survey study confirmed that many ICUs use aerosolized antibiotics, with some degree of ambivalence and with variable degrees of knowledge about how to optimize delivery (18). In the survey of 192 ICUs, the investigators found that 87 (45.3%) had some experience with intratracheal antibiotic administration, but 43.7% of healthcare workers did not use this therapy, mostly (in 78.6%) because of a lack of evidence–based guidelines recommending their use. There was some relationship between degree of experience with this therapy and the type of nebulizer used, with vibrating mesh nebulizers generally being used by those with the most experience. Less than 30% of all ICUs adhered to practices of efficient nebulizer use, indicating a great opportunity to improve how this methodology is applied in practice.

## Modern Aerosol Delivery Systems (Table 1)

Given the data of potential benefit of aerosolized antibiotics for ICU patients with VAP, it is important to assure optimal delivery to get the highest levels possible into the distal lung, in the areas of infection, and to minimize adverse effects. As is clear from the prior discussion, there is great variability in current clinical practice. In the survey study mentioned above, jet nebulizers were used in 38–49% of ICUs, ultrasonic nebulizers in about 35%, vibrating mesh nebulizers in < 10%, and still 12% were doing direct tracheal instillation of antibiotic solutions (18). In addition, only a little more than half the ICUs used gas delivery integrated with the ventilator, while the others used an external gas source.

**Table 1:** Nebulizer characteristics.

	**Aerosol generation**	**Ventilator settings**	**Placement in circuit**	**Comments**
Ultrasonic	High frequency piezo-electric Crystal in drug solution	Remove HME; turn off heated humidifier	15–40 cm upstream of Y connector	May overheat the drug solution; medium residual volume
Jet	Venturi effect on compressed gas	Remove HME; turn off heated humidifier	15–40 cm upstream of Y connector	Large residual volume; can interfere with ventilator
Vibrating mesh	High frequency mesh vibration in drug solution	Remove HME; turn off heated humidifier. Can run continuously or be breath actuated; optimize delivery with continuous flow by using 8 ml/kg, 50% duty cycle, respiratory rate of 12, inspiratory flow < 30 L/min, end inspiratory pause of 20%	Just proximal to Y connector; or distal to Y connector with breath actuated system	<5 micron particles, >60% reaches patient; no heating of solution during administration; leave the least amount of residual volume

Antibiotics can be administered as either a dry powder or liquid aerosol, although most clinical studies have been with liquid preparations. Ideally, any nebulized liquid solution should be prepared specifically for aerosol delivery, since aerosolization of intravenous preparations of antibiotics may contain chemicals in the delivery vehicle, that are irritating to the respiratory mucosa and can cause bronchospasm. No study has shown an advantage of ultrasonic, jet or vibrating mesh nebulizers in ICU patients (19, 20). Jet nebulizers generate aerosols by using compressed gas, ultrasonic nebulizers use a piezoelectric crystal to agitate a solution at high frequency, while mesh nebulizers use a high–frequency vibrating mesh to pump a solution through tapered holes (20, 21). In general, ultrasonic and vibrating mesh nebulizers are preferred over jet nebulizers, but the ultrasonic devices can overheat the antibiotic solution. In general, vibrating mesh devices produce particles < 5 microns in size, and up to 60% of the reservoir dose reaches the endotracheal tube, but it is uncertain how much reaches the pneumonic lungs. They are also smaller than other nebulizers and leave the least amount of residual solution at the end of the delivery process. For ventilated patients, the nebulizer is usually positioned in the inspiratory limb, before the Y–connector, and the HME filter is removed. If nebulization exceeds 30 min, humidification may need to be added. Unless a specialized delivery system is used, Rouby et al. have suggested that ventilated patients receive the nebulization with the ventilator set in the control mode, with the patient sedated, using a tidal volume of 7–9 ml/kg, a constant inspiratory flow and a minute ventilation < 6 L. Typically the respiratory rate is set at 12/min, the inspiratory: expiratory ratio at 1:1, with an end inspiratory pause of 20% of the duty cycle (8). In one recent clinical trial, a vibrating mesh nebulizer was placed proximal to the Y connector, and run continuously over 12 min with any ventilator having a bias flow < 4 L/min, and nebulization was left in place (2). In another large trial, a Pulmonary Drug Delivery System (PDDS) was utilized, with a vibrating mesh plate, and an electronic controller (22). The nebulizer was placed between the Y connector and the endotracheal tube, and connected electronically to a PDDS sensor in the inspiratory limb. The sensor led to activation of a control module that generated an aerosol during 25 to 75% of the inspiratory cycle, thus allowing air to start flowing before nebulization, and a wash in to occur after the nebulizer was stopped. Using this system, with a dose of amikacin 400 mg twice daily, most patients, achieved local antibiotic concentrations that exceeded the minimum inhibitory concentration (MIC) of the most resistant gram-negatives by a factor of at least 25 (22). With this system, drug delivery was not affected by humidification.

In ventilated patients, it is essential to optimize the method of nebulization, and it is possible that some of the early trials that failed to show benefit were influenced by a lack of stringent control over drug delivery methods. None the less, it still remains unclear if we are able to use nebulizers to deliver antibiotics to the site of infection, even if we are delivering it to the lung, in general. While some failures in clinical studies may be related to using inaccurate delivery systems, even modern delivery systems may fail to penetrate deeply into a consolidated lung. If the lungs have asymmetric areas of infection and mucus plugging, it is possible that drug is being delivered to healthy lung, but not to diseased lung, distal to dense airway obstruction by infected secretions.

## Recent Clinical Data with Aerosolized Antibiotics in the ICU (Table 2)

Three recent prospective randomized controlled trials of inhaled antibiotics for mechanically ventilated patients with pneumonia, have been completed, and the two largest have had negative results (2, 3).

**Table 2:** New studies of inhaled antibiotics for ICU pneumonia.

**Parameter**	**Phase II Inhaled amikacin trial (22)**	**Amikacin/Fosfomycin trial (2)**	**Inhaled vs. aerosol adjunctive amikacin (23)**	**INHALE trial [(3), NCT01799993]**
Design	Multicenter, randomized, double-blind, placebo-controlled	Multicenter, randomized, double-blind, placebo-controlled	Single-center randomized, controlled, not blinded	Multicenter, randomized, double-blinded, placebo-controlled
Number enrolled	69	143	133 post cardiac surgery patients	712
Intervention therapy	Inhaled amikacin 400 mg bid (*n =* 21), inhaled amikacin 400 mg daily (*n =* 26), inhaled placebo (*n =* 22) for 7–14 days; standard of care systemic antibiotics	Inhaled amikacin 300 mg/fosfomycin 120 mg bid (*n =* 71) vs. Placebo (*n =* 72) for 10 days, with intravenous meropenem or imipenem	Inhaled amikacin 400 mg bid plus systemic piperacillin-tazobactam (*n =* 86) vs. intravenous amikacin 20 mg/kg daily plus systemic piperacillin-tazobactam (*n =* 47). Duration dependent on patient response	Inhaled amikacin 400 mg bid (*n =* 354) vs. Inhaled placebo (*n =* 358) for 10 days; standard of care systemic antibiotics
Delivery device	Vibrating mesh nebulizer with PDDS system, distal to Y connector	Vibrating mesh nebulizer, used continuously, proximal to Y connector	Pneumatic nebulizer for ventilated patients; ultrasonic nebulizer for non-ventilated patients	Vibrating mesh nebulizer with PDDS system, distal to Y connector
Adjunctive vs. Salvage	Adjunctive, with high suspicion of gram-negatives	Adjunctive, with high suspicion of gram-negatives	Adjunctive aerosol vs. adjunctive IV	Adjunctive in patients with high suspicion of gram-negatives
Primary endpoint	Percent achieving tracheal amikacin concentration >6,400 mcg/ml	Change of CPIS from baseline, during therapy in patients with proven gram-negative infection	Clinical cure on day 7 of therapy	Mortality 28–32 days post start of therapy
Findings	50% achieved primary endpoint with amikacin 400 mg bid	No difference in CPIS during therapy (*p* = 0.7)	Higher clinical cure rate with nebulized therapy (91.8 vs. 70.2%) (*p* = 0.002)	No difference in mortality
Other findings	Reduced mean number of antibiotics per patient per day, at end of aerosol therapy; 0.9 with q12h, 1.3 with q24h, and 1.9 with placebo (*p* = 0.02)	No difference in mortality, clinical cure; higher rate of negative tracheal cultures for gram-negatives at day 3 and 7 with aerosol therapy	Nebulized therapy with: shorter time to clinical cure, shorter length of stay, less nephrotoxicity, less duration of amikacin, NO change in mortality	No difference in percent with early clinical response, days on ventilation, days in ICU, adverse events. More bronchospasm with inhaled therapy.

Kollef et al. conducted a randomized, placebo- controlled study of adjunctive (in addition to standard of care antibiotics) inhaled amikacin/fosfomycin (AFIS) (300 mg amikacin, 120 mg fosfomycin inhaled every 12 h for 10 days) in 143 patients with gram-negative VAP (2). The primary endpoint was change from baseline in the Clinical Pulmonary Infection Score (CPIS) during the 10 day course of therapy and no difference was seen with the use of adjunctive aerosolized antibiotics. Similarly, there was no difference in mortality or clinical cure. In the study, the aerosolized therapy was delivered by a vibrating mesh nebulizer, run continuously for 12 min, and placed proximal to the Y connector, using a preparation that was formulated specially for aerosol delivery. Humidification was allowed during administration, and there was no standardization of ventilator modes or ventilator settings. The most commonly identified pathogens were *Acinetobacter baumannii* and *P. aeruginosa***, **with 93% of the *Acinetobacter* being colistin and carbapenem resistant.

Although the trial was negative for the major endpoints, subgroup analysis gave some insights into the limitation of study design and the potential benefits to be explored in any subsequent studies. There was a significant reduction in the rate of positive tracheal cultures for gram-negative bacteria at day 3 and day 7 in the AFIS groups, compared to placebo, but again this led to no clinical differences, and the differences may have been affected by the physical presence of high concentrations of antibiotics (from inhalation) in the tracheal secretions of the AFIS patients. There were 13 pan drug-resistant (PDR) organisms, all *Acinteobacter baumanni*. In this group, clinical cure rates and ventilator free days trended in favor of the AFIS therapy group. One problem with the study was the use of prolonged duration of prior antibiotic therapy, before starting aerosol, often to treat non-pneumonia infections. Overall, the median duration of prior antibiotic therapy was 6 days in the AFIS group and 4 days in the placebo group. This may have masked some of the benefit of aerosol therapy, and in the cohort from the US, where prior therapy was for a median of 3 days, there a significant difference in change in CPIS, but not in mortality or clinical cure, with the aerosol therapy.

A single center trial, with a different design reported positive results (23). This trial by Hassan et al. studied 133 post cardiac surgery patients with microbiologically confirmed VAP or hospital acquired pneumonia, due to MDR gram–negative pathogens. All patients received intravenous piperacillin /tazobactam and then they were randomized to receive either intravenous amikacin, or aerosolized amikacin (400 mg twice daily). The study was not blinded, and little specific information is given about the nebulization procedure, other than to say that they used a simple pneumatic nebulizer in ventilated patients and an ultrasonic nebulizer in those with hospital-acquired pneumonia. However, the nebulized therapy led to significantly higher clinical cure rate, more rapid clinical cure, shorter duration of mechanical ventilation and shorter duration of amikacin therapy. The difference in clinical cure rates in favor of the aerosol therapy, did not apply to any specific pathogens. In spite of these benefits, there was no difference in mortality between the groups. There was however, less nephrotoxicity with the inhaled, compared to systemic amikacin therapy, and few patients had any adverse respiratory effects (bronchospasm) from aerosol therapy.

The largest study to date, the INHALE trial, compared adjunctive inhaled amikacin to inhaled placebo, in a randomized, blinded study of 712 patients with ventilated gram-negative pneumonia, and demonstrated no impact on mortality at 28–32 days after the start of therapy, the primary endpoint [(3), NCT01799993]. The trial was a phase III follow up of a prior, smaller, but successful phase II study. In the earlier study, 69 mechanically ventilated patients with gram-negative pneumonia, a CPIS score >6, at risk for infection with MDR pathogens, were randomized to adjunctive therapy with one of two doses of inhaled amikacin (400 mg once or twice daily), or placebo, for 7–14 days, using the PDDS delivery system discussed above (22). Half of the patients were infected with either *Acinetobacter baumannii* or *P. aeruginosa*. Aerosol therapy led to less antibiotic escalation with less addition of antibiotics, and a lower number of systemic antibiotics per patient per day at the end of therapy, when the high dose was compared to the lower dose and to the placebo.

As reported on clinical trials. gov, a total of 712 patients were enrolled in the INHALE study, with 354 receiving inhaled amikacin 400 mg every 12 h for 10 days, and 358 receiving inhaled placebo, using the PDDS aerosol system [(3), last accessed January 26, 2019]. Almost half of all patients had an APACHE II score > 20 and the mean CPIS score was 7 for all patients. Mortality was identical in the two groups (25.1 vs. 22.5% for amikacin vs. placebo), and secondary endpoints did not differ either. These included early clinical response, days on mechanical ventilation (10–11 days), days in ICU (21–22 days), with similar rates of adverse events (although there was a trend to more bronchospasm in the inhaled antibiotic group). This study is yet to be reported in full detail in a peer-reviewed publication.

## Why Have Inhaled Antibiotic Trials Failed, and Should this Therapy be Abandoned?

Given the negative results of two large randomized controlled trials, it is important to consider if they failed because the concept of benefit from routine adjunctive inhaled antibiotic therapy for gram-negative VAP is not correct, or if there were features of trial design that limited the likelihood of the trials' success.

For trial design, it is unlikely that inhaled therapy will reduce mortality in ICU patients, since this endpoint is subject to multiple influences, in addition to the efficacy of pneumonia therapy, as was the case in the INHALE study (3). Although clinical cure would be a reasonable target for study outcome, this is relatively subjective, and would only have value in a blinded study. Although the CPIS can be viewed as a more numerical endpoint, it too is subjective, particularly with regard to assessing purulence of secretions. As discussed, the change in CPIS during therapy was not affected by the use of aerosolized amikacin/fosfomycin (2). A microbiologic eradication endpoint is not a good target, since it may not be relevant if not related to an improved clinical outcome, and it may be further impacted by the physical presence of antibiotics in cultured secretions, leading to a false sense of microbiologic success. An endpoint of reducing the use of systemic antibiotics would be valuable to patients and to ICU care, and the phase II study of inhaled amikacin did find that using adjunctive aerosolized therapy reduced the total amount of systemic antibiotics, compared to placebo (22). The problem with this as an endpoint, is that it would have to be used in the context of a short duration of standard therapy. If all patients routinely received 14 or more days of systemic therapy, regardless of clinical response, any benefit of aerosol therapy could be masked. However, new guidelines recommend 7–10 days of therapy for VAP, and if adjunctive aerosol were used with this duration of systemic therapy, allowing longer for non-responders, it is possible that adjunctive aerosol therapy could reduce the amount of systemic antibiotics prescribed (24, 25). This in turn could lead to benefits in secondary endpoints such as antibiotic side effects and emergence of resistance during therapy. In fact, Palmer et al. did find that adjunctive aerosolized antibiotics led to less resistance compared to using only systemic antibiotics in a small double blinded study of patients with either VAP or ventilator-associated tracheobronchitis (26). We may also need to have studies of exclusively inhaled antibiotic therapy for patients with MDR pathogens, resistant to all systemic antibiotics, compared to best available systemic therapy.

Patient selection is also key in trial design. Should all VAP patients be included, or only those with proven or highly suspected MDR gram negative pneumonia? Should therapy be given empirically and as an adjunct to systemic therapy in at risk patients, or should therapy be reserved only when MDR pathogens are found, or when patients are failing systemic therapy (as recommended in some current guidelines, as discussed below)? Most trials have focused only on MDR gram-negatives, but the study by Palmer et al. included inhaled aminoglycosides and inhaled vancomycin as an option to treat gram-positive organisms as well (10). Another consideration is whether therapy should be limited to patients who are mechanically ventilated at the time of pneumonia diagnosis, or if non-ventilated patients should also be treated. It is also important to limit the duration of systemic antibiotics prior to enrollment. As was seen in the amikacin/fosfomycin trial, many patients had a prolonged course of systemic therapy prior to aerosol, and this may have masked a benefit, since those with fewer days of prior therapy appeared to show improvement with aerosol therapy, compared to placebo (2). Finally, it would be important to enroll patients with a limited range of illness severity, or APACHE II score. Ideally patients should have a score between 10 and 25 since those with minor illness may improve regardless of therapy, and those with very severe illness may be unable to respond, even to effective therapy.

As has been discussed, it is also important to optimize aerosol delivery in ventilated patients. This will require use of a reliable particle nebulizer, probably a vibrating mesh device, along with proper synchronization with the ventilator cycle. Careful attention to delivery has not been done in many older studies, and this lack of uniformity makes the therapy potentially ineffective. It is also important to define the proper duration of adjunctive aerosol therapy, but it probably should parallel systemic therapy, and be for 7–10 days. Without this standardization, it is impossible to know if enough drug reached the lower respiratory tract, and even if it did, it is important to monitor drug levels. In the phase II inhaled amikacin study only 50% of patients getting amikacin 400 mg twice daily achieved a tracheal aspirate amikacin concentration >6,400 micrograms/ml, which was 25 times the minimum inhibitory concentration of the most resistant pathogens (22). Even in this setting, there is some concern that areas of pneumonic consolidation may not see this much antibiotic, due to mucus obstruction of the airways leading to the infected lung. It is possible, that over several days, as the consolidation resolves, more and more aerosolized drug is able to penetrate distally.

It is likely that inhaled therapy will be useful, based on some of the positive preliminary data, but success will be highly dependent on trial design, as summarized in Table 3. In most experimental and clinical studies, lung tissue concentrations in infected lung, as well as clinical efficacy, were assessed by continuous delivery nebulizer systems. Nebulization that is synchronized with inspiration would be ideal, but these devices are not yet commercially available, and comparison between continuous and synchronized systems still needs to be done. In future large clinical trials, it is essential to optimize nebulization techniques for drug delivery efficiency and alveolar drug concentrations.

**Table 3:** Features to consider in the design of future trials of inhaled antibiotics.

**Trial design**
Clinical endpoint
Mortality
Clinical cure
Microbiologic eradication
Change in CPIS over time
Reduction in use of systemic antibiotics and antibiotic side effects
Other endpoints: Monitor for emergence of resistance during therapy
Design features
Adjunctive to systemic therapy routinely, or only as salvage therapy?
Limit prior systemic antibiotics
Define duration of systemic therapy for pneumonia: 7–10 days maximum
Define duration of inhaled antibiotics: to parallel intravenous antibiotics
Antibiotic choice
Only gram–negatives or also include therapy for gram-positives (use more than one drug)?
Define dose of inhaled antibiotic
Specify which systemic therapy is used.
**Patient population**
Ventilated only, or also include non-ventilated?
At risk for MDR pathogens or proven MDR pathogens?
Only MDR gram–negatives or also MDR gram–positives?
APACHE II of 10–25
**Technical considerations**
Define aerosol delivery device
Define how to coordinate device with ventilator
Define ventilator settings, sedation, humidification
Measure drug levels in tracheal secretions and in distal lung lavage from pneumonic and non-pneumonic areas

## Current Recommendations

Currently, the Infectious Diseases Society of America/ American Thoracic Society guidelines for nosocomial pneumonia recommend (weak recommendation, low quality of evidence) the addition of inhaled antibiotics to systemic antibiotics for patients with gram-negative pneumonia due to MDR pathogens, sensitive only to polymyxins, and aminoglycosides (24). They recommended the use of colistin inhaled rather than polymyxin B. They also suggested that this therapy could be used as a last resort for non-responding VAP patients, with sensitive or resistant pathogens. In contrast, the European nosocomial pneumonia guidelines did not comment on the use of inhaled antibiotics until more data showing efficacy were available (25). Currently, there are no recommendations for using inhaled antibiotics as a routine adjunctive therapy, even for high risk patients, but some data support their use only after culture data are available showing MDR pathogens, or as a form of salvage therapy (1). At the current time, our practice is to use inhaled adjunctive therapy for patients with highly resistant gram-negative pathogens, along with systemic therapy, using a vibrating mesh nebulizer, synchronized with the inspiratory cycle of the ventilator. We treat generally for 10 days for patients with these MDR pathogens, and follow clinical endpoints to decide when to stop therapy. We do not currently use routine empiric adjunctive aerosolized therapy. However, with future trials, if properly designed, the benefits of routine adjunctive therapy, particularly for endpoints related to reducing systemic antibiotic use may emerge.

## Author Contributions

The author confirms being the sole contributor of this work and has approved it for publication.

### Conflict of Interest Statement

MN was an investigator in the INHALE trial (3) and a consultant to Bayer for this trial.
